# Therapeutic Potential of Heme Oxygenase-1 and Carbon Monoxide in Acute Organ Injury, Critical Illness, and Inflammatory Disorders

**DOI:** 10.3390/antiox9111153

**Published:** 2020-11-19

**Authors:** Stefan W. Ryter

**Affiliations:** Joan and Sanford I. Weill Department of Medicine, Weill Cornell Medical College, 525 East 68th Street, Room M-522, Box 130, New York, NY 10065, USA; str2020@med.cornell.edu

**Keywords:** acute lung injury, acute respiratory distress syndrome, carbon monoxide, heme oxygenase, ischemia/reperfusion injury

## Abstract

Heme oxygenase-1 (HO-1) is an inducible stress protein that catalyzes the oxidative conversion of heme to carbon monoxide (CO), iron, and biliverdin (BV), the latter of which is converted to bilirubin (BR) by biliverdin reductase. HO-1 has been implicated as a cytoprotectant in various models of acute organ injury and disease (i.e., lung, kidney, heart, liver). Thus, HO-1 may serve as a general therapeutic target in inflammatory diseases. HO-1 may function as a pleiotropic modulator of inflammatory signaling, via the removal of heme, and generation of its enzymatic degradation-products. Iron release from HO activity may exert pro-inflammatory effects unless sequestered, whereas BV/BR have well-established antioxidant properties. CO, derived from HO activity, has been identified as an endogenous mediator that can influence mitochondrial function and/or cellular signal transduction programs which culminate in the regulation of apoptosis, cellular proliferation, and inflammation. Much research has focused on the application of low concentration CO, whether administered in gaseous form by inhalation, or via the use of CO-releasing molecules (CORMs), for therapeutic benefit in disease. The development of novel CORMs for their translational potential remains an active area of investigation. Evidence has accumulated for therapeutic effects of both CO and CORMs in diseases associated with critical care, including acute lung injury/acute respiratory distress syndrome (ALI/ARDS), mechanical ventilation-induced lung injury, pneumonias, and sepsis. The therapeutic benefits of CO may extend to other diseases involving aberrant inflammatory processes such as transplant-associated ischemia/reperfusion injury and chronic graft rejection, and metabolic diseases. Current and planned clinical trials explore the therapeutic benefit of CO in ARDS and other lung diseases.

## 1. Introduction

**Figure 1:**
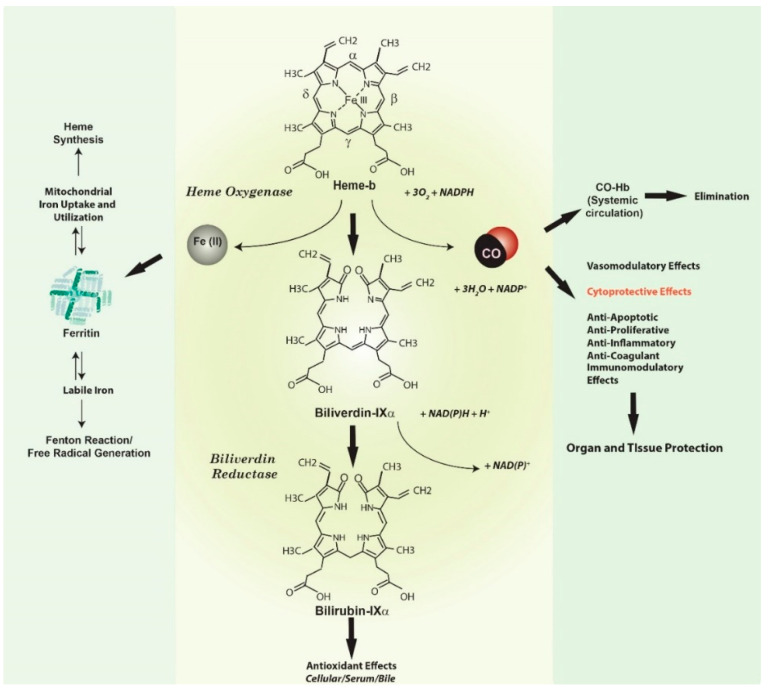
**Heme oxygenase activity and end-product generation.** Heme oxygenase (HO) is a metabolic enzyme that catalyzes the rate-limiting step in the oxidative catabolism of heme to generate the bile pigment biliverdin-IXα (BV) [1,2]. The HO reaction requires molecular oxygen (O_2_) and reducing equivalents from NADPH cytochrome p-450 reductase. The HO reaction releases the α-methene bridge carbon of heme as carbon monoxide (CO). The heme iron is released as ferrous iron (Fe II). BV is further metabolized to bilirubin-IXα (BR) by NAD(P)H biliverdin reductase [1,2,3]. Both BV and BR are potent antioxidants [22,23,24]. The metabolic fate of iron released by HO activity remains incompletely understood. Iron may act as a catalyst for the Haber-Weiss cycle, leading to the production of the highly reactive and deleterious hydroxyl radical, and may promote other free radical generating reactions such as the decomposition of organic peroxides [27]. Seminal studies suggested that HO-derived iron can trigger the synthesis of ferritin via modulation of iron binding protein activity [21]. Ferritin can in turn act as a potential cytoprotectant in the endothelium. Endogenously produced HO-1 derived CO has a myriad of possible biological effects, including vasoregulation, as well as cytoprotective effects that include regulation of inflammation, apoptosis, and cell proliferation, and modulation of immune responses [17,18,25,26].

***Heme oxygenase-1, a vital heme degradative enzyme and inducible stress protein***. Heme oxygenase (HO; E.C. 1:14:99:3) is a metabolic enzyme system first described in the late 1960s which provides the rate-limiting step in the oxidative degradation of heme [1,2]. Heme catalysis by HO requires molecular oxygen (O_2_) and NADPH: cytochrome p-450 reductase as the electron source [1,2] (Figure 1). The HO reaction generates several products, each with biological sequelae, including carbon monoxide (CO) (which evolves from the α-methene bridge carbon of heme), the bile pigment biliverdin-IXα (BV), and free ferrous iron, Fe(II). BV is subsequently converted to its lipid soluble metabolite bilirubin-IXα (BR) by cytosolic NADPH: biliverdin reductase (BVR) [3].

Heme degradative activity is catalyzed by two major isoforms of HO, a constitutive form (heme oxygenase 2, HO-2) and an inducible form (heme oxygenase 1, HO-1), which are products of distinct genes [4,5]. The enzymatic HO substrate, heme, provides prosthetic groups for hemoglobin, the principal O_2_ carrier in the blood, and serves as a cofactor for mitochondrial cytochromes and other metabolic enzymes [6,7]. Heme, in free form, may also represent a hazardous pro-oxidant compound due to the presence of the central iron, a potential catalyst of harmful free radical generating processes [8]. Indeed, heme accumulation has been implicated as a pathological mediator in various inflammatory conditions such as sepsis [9,10,11], malaria [12], sickle cell disease [13], and atherosclerosis [14]; with its clearance by HO-1 implicated as a protective and anti-oxidative strategy in these and other diseases.

HO-1 is not only recognized as an essential metabolic enzyme, but also as a key intermediate in the stress response and in cellular adaptation to injury [15]. The upregulation of HO activity, specifically via the transcriptional regulation of HO-1, has been widely associated with cytoprotection and the protective dampening of inflammation in many cellular and preclinical models of injury [16,17,18].

Under conditions where HO-1 is found to be associated with cytoprotection, a plausible hypothesis is that the individual HO reaction products contribute, individually or collectively, to the implicit cytoprotective properties of HO-1 [19]. This hypothesis does not exclude activity-independent functions of the protein, such as potential intramolecular interactions resulting in the regulation of other proteins [20]. The iron release from the HO reaction drives the synthesis of ferritin, an iron sequestering macromolecule that may contribute to cytoprotection in vascular endothelium and other cell types [21]. Both BV, and its metabolite BR, are known to exert natural antioxidant properties in serum and bile [22,23,24]. CO evolution may contribute to the modulation of cellular function resulting in cellular adaptation, and this notion has provided a rationale for the application of pharmacological or gaseous CO to harness its therapeutic potential [25,26]. In contrast, pro-pathogenic and detrimental roles of excess HO-1 activation have also been proposed [27,28]. For example, the cytoprotective properties of HO-1 may confer a survival advantage to tumor cells, and thus contribute to cancer progression [29]. In neurodegenerative diseases, the excessive release of pro-oxidant iron by HO activity may contribute to neural injury and disease pathogenesis [30].

The crucial role of HO-1 in systemic homeostasis, has been uncovered by rare cases of HO-1 deficiency, which have been associated with endothelial cell injury, anemia, abnormal tissue iron accumulation, inflammation, and nephritis [31,32]. HO-1 knockout mice (*Hmox1*^-/-^) show evidence of hepatic and renal iron deposition, serum anemia, and increased vulnerability to oxidative stress [33]. HO-1 is now recognized as a ubiquitous stress protein whose transcriptional regulation can be induced in cells and tissues by numerous environmental toxins and exogenous effectors [16,34]. These include both the enzyme substrate heme and its downstream catabolic product CO, physical stresses (i.e., heat shock and ultraviolet-A radiation), as well as chemical agents such as heavy metals, thiol (-SH) reactive substances, nitric oxide (NO), natural or synthetic electrophilic antioxidant compounds (e.g., curcumin, resveratrol), high and low oxygen tension (i.e., hyperoxia, hypoxia), pro-inflammatory and pro-oxidant states, pro-inflammatory cytokines, and hormones [18]. The transcriptional regulation of HO-1, while influenced by multiple nuclear factors is primarily governed by nuclear factor erythroid 2–related factor 2 (Nrf2), a master regulator of the cellular antioxidant response, which may act in combination with cell-and inducer-specific accessory transcription factors [35,36].

Therapeutic targeting of HO-1 has been proposed for a number of diverse diseases and conditions, and these include neurological diseases [37], acute lung injury (ALI) [38], acute kidney injury (AKI) [39,40,41], ischemia/reperfusion injuries (IRI) of multiple organs, including lung, kidney, heart, liver, and others [42,43,44], cardiovascular diseases [14,39,45,46], metabolic diseases [47,48,49,50], and cancer [29,51,52], as described in recent reviews. In many of these indications, therapeutic application of CO, using either gas or the pharmacological application of CO-releasing molecules (CORMs), have also been demonstrated [17,25]. This review will focus on therapeutic implications of HO-1/CO in organ injury and inflammatory diseases, as they relate to disorders of pulmonary and critical care medicine, such as acute organ injuries and acute respiratory distress syndrome (ARDS), pneumonias, sepsis, and related conditions. 

***Heme oxygenase-derived carbon monoxide is a potential physiological mediator***. CO, a low molecular weight gas, has been widely studied in the global biomedical research community as a cytoprotective and homeostatic molecule with important signaling capabilities in physiologic and pathophysiologic contexts. CO entering the bloodstream forms carboxyhemoglobin (CO-Hb) due to its high affinity for hemoglobin heme in competition with O_2_, which in turn impairs O_2_ delivery to tissues. Early studies have suggested a candidate signal transduction role for HO-2 derived CO in olfactory neurotransmission [53]. CO has also emerged as a novel gaseous therapeutic molecule in the context of various experimental and human diseases. It has been known since the mid-twentieth century that CO is endogenously produced in man in the natural course of systemic hemoglobin turnover, and as the by-product of cellular heme and hemoprotein turnover [54,55,56]. In this regard, the endogenous production of CO primarily occurs as the product of heme degradative enzymatic activities (i.e., HO-1, HO-2) [57].

The recognition of the cytoprotective nature of HO-1 led to extensive investigation of CO as a potential mediator of this effect. These investigations precipitated studies in the use of exogenous CO as a modulator of cellular function and adaptive responses. In seminal studies by Otterbein et al., exogenous CO was found to limit macrophage inflammatory responses in a manner similar to HO-1 expression and to provide anti-inflammatory protection in models of hyperoxia-induced ALI [58,59,60]. This work later uncovered that CO can potentially mitigate inflammatory injury via a number of cellular mechanisms, which include modulation of classical Toll-like receptor (TLRs)-dependent pro-inflammatory cytokines/chemokines production [58,61,62], modulation of inflammasome activation and its regulated cytokines [63,64], modulation of regulated cell death programs (i.e., apoptosis) [65,66], modulation of cellular autophagy (a cellular homeostatic program for protein and organelle turnover) [67], and the regulation of the resolution phase of inflammation [68,69,70] (see refs. [17,26] for reviews).

**Figure 2:**
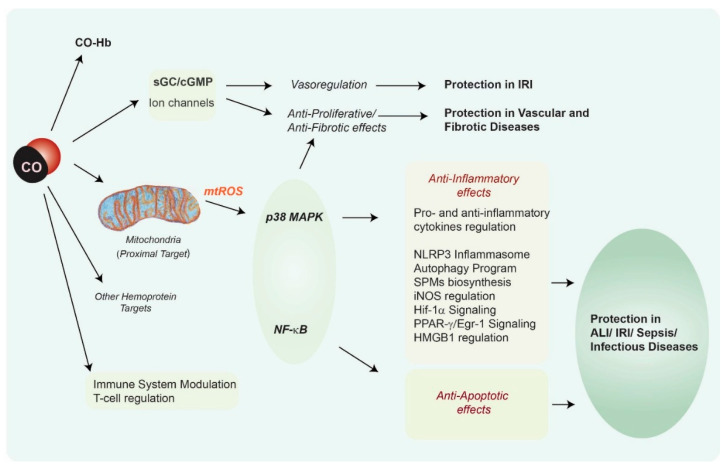
**Signaling targets of CO**. CO has a high affinity for iron centers and thus can bind and modulate the cellular activity of hemoproteins. CO has been proposed to target sGC, which is a primary receptor of, and has a higher affinity for, nitric oxide [71]. The vasoregulatory effects of exogenous CO, are likely modulated by sGC, with possible contribution from direct ion channel modification [26]. CO has been implicated in several cellular effects, which include the modulation of inflammation, apoptosis and other cell death programs, and cellular proliferation. CO can also target immune responses by altering T cell proliferation and differentiation [17]. Mitochondria, the site of cellular energy generation, are believed to represent primary targets of CO action [72,73,74,75,76,77,78] (see Figure 3 for detail). CO can modulate inflammatory responses via mtROS [63,74,75,76,77,78] and/or p38 MAPK-dependent inhibition of pro-inflammatory cytokines (e.g., IL-1β, TNF-α) and upregulation of the pro-inflammatory cytokine IL-10 [58,60]. The anti-inflammatory effect of CO has been associated with the modulation of Toll-like receptor activation [61,62]. The modulation of p38 MAPK and NF-κB activities also can modulate the apoptosis program in response to pro-death stimuli [65,66]. Additional mechanisms have been implicated in the regulation of inflammation by CO, including PPARγ/Egr-1 signaling [73], modulation of hypoxia-inducible factor-1alpha (Hif-1α) signaling [77], and normalization of HMGB1 levels [79]. CO can regulate the NLRP3 inflammasome pathway, a specialized platform for the maturation and secretion of pro-inflammatory cytokines such as IL-1β and IL-18. CO can also modulate the synthesis of specialized pro-resolving mediators, SPMs, to promote the resolution of inflammation [68,69,70]. Abbreviations: cGMP: cyclic 3′-5′ guanosine monophosphate; CO: carbon monoxide, EGR-1: Early growth response-1; Hif-1α: hypoxia-inducible factor-1alpha; HMGB1: high mobility group box-1; MAPK: mitogen-activated protein kinase; NF-κB: nuclear factor kappa-B; NLRP3: nucleotide-binding domain, leucine-rich-containing family, pyrin domain-containing-3; PPAR-γ: peroxisome proliferator-activated receptor-γ; sGC: soluble guanylate cyclase; SPMs, specialized pro-resolving mediators.

***Cellular targets of CO: From mitochondria to the modulation of signal transduction pathways.*** Cumulative research studies over the last two decades have revealed that CO can regulate cellular processes (including anti-inflammatory and tissue protective effects) via modulation of cellular signaling targets. These key pathways and mediators include soluble guanylate cyclase (sGC), a known target of NO, which generates the second messenger cyclic 3′-5′ guanosine monophosphate (cGMP) upon gaseous ligand binding. The heme of sGC has a far greater affinity for NO than CO. Thus, the competition of CO for sGC is of unclear physiological significance but may be relevant under conditions of reduced NO bioavailability or therapeutic application of CO to the lung [71]. Secondary targets and effector molecules activated by CO have also been identified, most notably mitogen activated protein kinases (MAPKs), and nuclear factor kappa-B (NF-κB) [25,26]. These mechanisms are summarized in Figure 2. Since CO binds primarily to iron containing prosthetic groups, mainly represented by heme, and has little biological reactivity outside this sphere, primary CO sensing targets are likely represented by cytochromes and other heme-containing proteins. This is exemplified by the classical target sGC, which contains heme, and other enzymes containing heme prosthetic groups such as inducible nitric oxide synthase (iNOS), cytochrome *c* oxidase, heme-containing transcription factors (e.g., Rev-Erb-α/β), and NADPH:oxidases (NOXs) which generate superoxide anion (O_2_^−^) for host defense.

**Figure 3:**
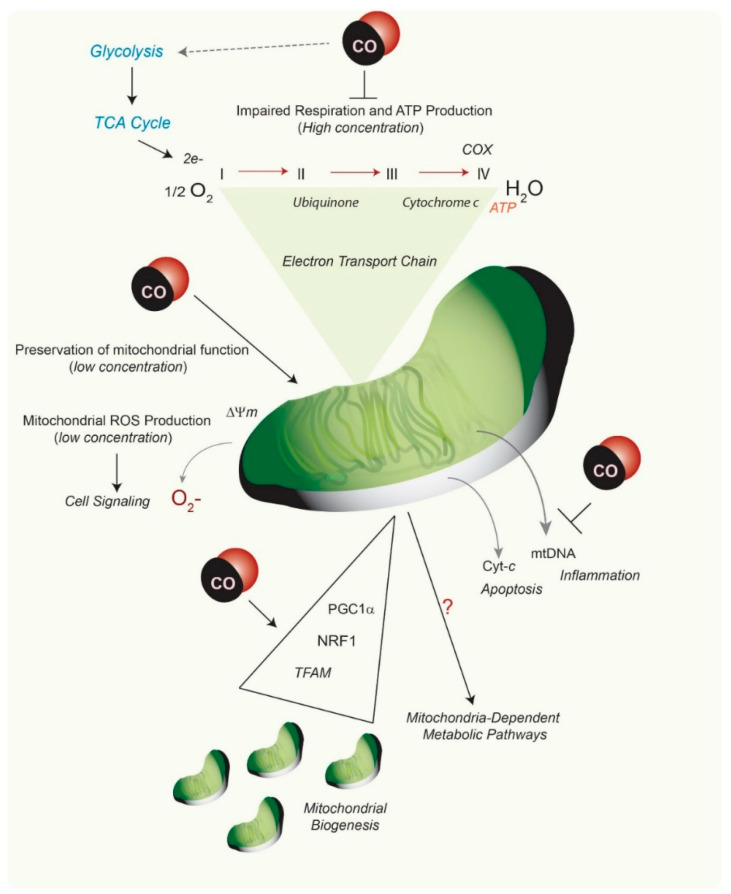
**Mitochondria as a target of CO action**. CO has been shown to preserve mitochondrial function at low concentration, including preservation of mitochondrial membrane potential which can be disrupted by pro-inflammatory stimuli [63]. Stimulation of various cell types with CO can modulate mitochondrial ROS (mtROS) production, in a cell type specific and context-specific manner [63,73,74,75,76,77,78]. CO can also modulate the activity of mitochondrial hemoproteins, including cytochrome c oxidase (COX). At high concentration CO can inhibit mitochondrial respiration, inhibit mitochondrial energy (ATP) production [71], and also modulate the glycolytic pathway in a dose-dependent manner [80,81]. CO has been implicated in several cellular effects, which include the modulation of apoptosis [65,66] and (inflammasome-dependent) inflammation [63,64], that are mediated by mitochondria-derived soluble factors, including cytochrome-c (Cyt-*c*) and mitochondrial DNA (mtDNA), respectively. Finally, CO has been shown to act as a potent modulator of mitochondrial dynamics and biogenesis in various studies [82,83,84,85,86,87,88]. These effects were contingent on the upregulation of regulatory factors: PPAR-γ coactivator-1α (PGC-1α), nuclear respiratory factor 1 (NRF1), and mitochondrial transcription factor A (TFAM) [87].

Mitochondria, key organelles responsible for cellular energy production, have been implicated as an important proximal target for CO-dependent regulation of cellular physiology and signaling (Figure 3). Mitochondrial reactive oxygen species (mtROS) arise as byproducts of ordinary metabolism, whose production can increase with respiratory chain inhibition or increased O_2_ tension, and which can contribute to physiologic or pathophysiologic signaling. Mitochondria are rich in iron centers, including the heme moieties of respiratory chain cytochromes, which have been implicated as binding targets for CO. CO is a well-known mitochondrial poison, whose exposure at high concentrations can impair mitochondrial respiration via binding to the cytochrome a3 subunit of cytochrome *c* oxidase [72]. CO exposure can influence mtROS production, mitochondrial membrane potential (Δ_Ψm_), and modulate mitochondria-dependent metabolic pathways [73,74,75,76]. In macrophages, the upregulation of mtROS by low concentration CO was associated with inhibition of cytochrome *c* oxidase, which promoted anti-inflammatory effects via p38 MAPK signaling. These studies reported a stabilizing effect of CO on cellular ATP production and Δ_Ψm_ [75]. Elevation of mtROS in macrophages by low-dose CO was shown to trigger adaptive cellular signaling through upregulation of peroxisome proliferator-activated receptor-γ (PPAR-γ) reduced expression of the pro-inflammatory factor early growth response-1 (Egr-1) [73], and the stabilization of hypoxia-inducible factor-1-alpha (HIF-1α), leading to coordinated anti-inflammatory effects [77]. CO, when administered in the absence of other stimuli, upregulated mtROS production in pulmonary epithelial cells, resulting in activation of cellular autophagy [74]. Administration of CO antagonized mtROS production in lung epithelial cells under hyperoxia, where ROS production was elevated due to high O_2_ tension and protected epithelial cells from hyperoxia-induced apoptosis [74]. CO impaired mtROS production in macrophages in the presence of inflammasome-activating stimuli such as LPS and ATP, a cellular model of nucleotide-binding domain, leucine-rich-containing family, pyrin domain-containing-3 (NLRP3) inflammasome activation [63]. Treatment with LPS and ATP caused mitochondrial depolarization and cytosolic translocation of mtDNA, a marker of mitochondrial injury, in bone marrow-derived macrophages, whereas CO exposure reduced these effects [63].

Studies using model CORM compounds, have also uncovered CO effects on mitochondrial stability and metabolism. A number of CORMs with different properties have been developed for experimental use, including ruthenium, molybdenum, and iron-based compounds (see Table 1). In isolated cardiac mitochondria, exposure to CORM-3 (Tricarbonylchloro(glycinato)ruthenium (II)) was shown to cause mitochondrial uncoupling. At low concentrations CORM-3 derived CO increased mitochondrial O_2_ consumption, but limited respiration at higher concentrations via inhibition of cytochrome *c* oxidase. CORM-3 also caused time-dependent decline of Δ_Ψm_ and increase of complex I-dependent mtROS production. In contrast, CORM-3 inhibited complex II-dependent mtROS generation in isolated mitochondria [78]. Recent studies using a manganese-based CORM (CORM-401) (Mn(CO)4(S2CNMe(CH2CO2H)) as a model CO donor compound reveal effects of CO on mitochondrial metabolism. Exposure to this compound caused a short-term shift from glycolytic metabolism to pentose phosphate pathway activation, with increased production of NADPH, and increased production of mtROS. Longer exposures of CORM-401 resulted in impaired mitochondrial respiration, loss of ATP production, increased glycolysis, and increased proton leakage [80]. The mitochondrial uncoupling effect of CORM-401, characterized by increased O_2_ consumption, was associated with activation of mitochondrial large-conductance calcium-regulated potassium ion channels [81].

Mitochondria are dynamic organelles subject to genetically regulated programs for their elongation, fragmentation, and biosynthesis. The effects of HO-1 or CO on mitochondrial dynamics (e.g., fusion, fission) have not been widely studied. Recent studies have described associations between the HO-1 and mitochondrial quality control in a rat model of sepsis [82]. HO-1 activation via heme-dependent stimulation of the PI3k/Akt pathway promoted mitochondrial quality control, including prevention of fission, and promotion of mitochondrial biogenesis, and activation of mitophagy [83]. The expression of mitofusin-1, a regulator of mitochondrial fusion, was associated with HO-1 activation in this model [83]. The incidence of mitochondrial fission, and excess mtROS production in the LPS model were also improved by application of CORM2 (tricarbonyldichlororuthenium(II) dimer) or hemin [84].

CO has been established as an endogenous and exogenous regulator of mitochondria biogenesis, a process that regulates de novo generation of mitochondria [85,86,87]. CO stimulation of cardiac mitochondrial biogenesis protected against doxorubicin-induced cardiomyopathy [86]. CO-dependent stimulation of cardiac and skeletal muscle mitochondrial biogenesis required increased PPAR-γ coactivator-1α (PGC-1α), nuclear respiratory factor 1 (NRF1), and mitochondrial transcription factor A (TFAM) expression. The induction of mitochondrial biogenesis by CO was also shown to require activation of the sGC/cGMP axis, the AKT pathway, and increased production of mtROS [87].

Recent studies have shown relationships between the ER stress response and regulation of mitochondrial biogenesis by CO. The activation of protein kinase R (PKR)-like endoplasmic reticulum (ER) kinase (PERK) by CO increased the nuclear translocation of transcription factor EB (TFEB) in cultured hepatocytes. Genetic deficiency of TFEB abrogated CO-dependent increase in mitochondrial biogenesis markers (PGC1α, NRF1, and TFAM), and mitochondrial proteins COX II, COX IV, and cytochrome *c* in hepatocytes. CO inhalation reduced liver injury in a mouse model of lipopolysaccharide (LPS)/D-galactosamine (D-GalN)-induced liver injury. Furthermore, CO inhalation increased TFEB activation, mitophagy, and mitochondrial biogenesis in mice treated with LPS/GalN [88]. Similarly, the application of CORM-3 prevented mitochondrial function decline and stimulated mtROS-dependent activation of mitochondrial biogenesis in septic mice [85].

**Table 1:** Representative CORM compounds.

CORM	Chemical Formula	Properties	Structure	Refs.
CORM2	Tricarbonyl dichloro Ruthenium (II) dimer. [Ru(CO_3_)Cl_2_]_2_	Fast CO release kinetics. Activatable by ligand substitution. Lipid/ DMSO soluble.	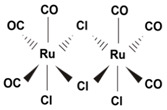	[89]
CORM3	Tricarbonylchloro (glycinato) ruthenium (II). C_5_H_4_ClNO_5_Ru	Fast CO release kinetics. Activatable by ligand substitution. Water soluble.	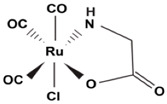	[78,89]
CORM-A1	Sodium boranocarbonate. CH3BNa_2_O_2_	Slow CO release kinetics. Activatable by pH changes. Water soluble.	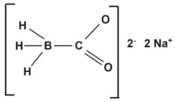	[90]
CORM-401	Tetracarbonyl[N-(dithiocarboxy-?S,?S′)-N-methylglycine] manganate. (Mn(CO)_4_(S2CNMe(CH_2_CO_2_H)))	Releases 3 mol CO per molecule. Activatable by oxidants, Soluble in DMSO.	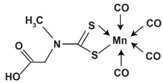	[81,91]
CORM-S1	Dicarbonyl-bis(cysteamine) iron(II). Fe(SCH_2_CH_2_NH_2_)_2_ (CO)_2_	Photoactivatableby visible light (>400 nm). Prototype “PhotoCORM”	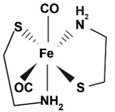	[92,93]

In conclusion, the effects of CO on mitochondrial respiration, mitochondrial membrane potential, and mtROS generation are clearly dose-dependent, and are responsible in part for triggering other downstream signaling sequelae leading to adaptive cellular responses. Furthermore, studies describing stimulation of mitochondrial biogenesis by CO, taken together, suggest a potential novel mechanism by which CO treatment can lead to adaptive responses to systemic stress.

## 2. Protective Roles of HO-1/CO in Inflammatory Conditions

The therapeutic rationale for applying inhaled CO (iCO) is based on numerous animal model studies (Table 2) [17,18,25,26]. iCO exerts anti-inflammatory effects in animal models of tissue injury and disease, specifically those involving inflammation and innate immune responses in the pathogenesis. Additionally, iCO has been shown to exert other protective effects in disease models, including inhibition of programmed cell death (apoptosis), inhibition of cellular proliferation and platelet aggregation, and vascular effects [17,18,25,26]. These salutary effects of iCO were demonstrated in models of inflammatory injury and disease, and in both rodents [58,59,60] and higher mammals such as swine and non-human primates (NHPs) [70,94,95,96]. In addition, recent studies have demonstrated that CO decreases inflammation, enhances phagocytosis and improves mortality in models of sepsis including endotoxemia [58], hemorrhagic shock [97,98,99], and cecal-ligation and puncture (CLP) [67,100], reduces lung injury incurred by mechanical ventilation [101,102,103], and reduces IRI in models of organ transplantation including the lung [104,105,106,107], kidney [108,109,110], and other organs [111].

***Endotoxemia/LPS-induced Inflammation:*** HO-1 has been identified as a mediator of the acute inflammatory response, as previously shown in animal models of inflammation and acute lung injury [58,68]. Early studies found that HO-1 expression by gene transfer protected against LPS-induced ALI in mice involving the increased production of the anti-inflammatory cytokine IL-10 [112], and also limited murine ALI following influenza virus infection [113].

**Table 2:** Representative studies showing CO-dependent protection in inflammation models.

Injury/Disease Model	Species	Dose	Observation	Ref.
LPS	Mice	250 ppm [100–500 ppm] CO	Anti-inflammatory effect, reduction of pro-inflammatory cytokines (TNFα, IL-6), p38 MAPK, JNK, IL-6 dependent. Upregulation of serum IL-10.	[58,114,115,116]
“	Pig	250 ppm	Reduced disseminated intravascular coagulation; Reduction of pro-inflammatory cytokines (IL-1β) and endothelial ICAM expression. Upregulation of IL-10	[94]
“	Macaque	500 ppm CO	Reduction of pro-inflammatory cytokine (TNFα), >30% CO-Hb	[95]
Pneumonia-ALI	Baboon	100–300 ppm CO	Reduction of lung inflammation, improved histology	[96]
Hyperoxia	Rat	250 ppm CO	Reduction of lung edema, inflammation, fibrin deposition, and apoptosis; improved histology.	[59]
“	Mice	250 ppm CO	Reduction of lung inflammation; p38 MAPK-dependent	[60]
VILI	Rat	250 ppm CO	Reduction of lung inflammation, Reduced neutrophil influx	[103]
“	Mice	250 ppm CO	Reduction of lung inflammation, Improved lung histology	[101,102]
MA-ALI/ECM	Mice	CORM (ALF-492 36.7 mg/kg) 50 ppm CO	Improved lung histology and survival/Protection in CM, reduced neuroinflammation.	[117,118,119,120,121]
Hemorrhagic shock	Mice	250 ppm CO CORM3 (4 mg/kg)	Reduced inflammation, apoptosis. Upregulation of IL-10.	[97,98,99]
Sepsis/CLP	Mice	250 ppm CO	Increased autophagy, improved bacterial clearance	[67,100]
“		CORM2 (30 mg/kg)	Reduced HMGB1 expression, Improved survival in sepsis	[79]
Lung I/R	Mice	250 ppm CO	p38 MAPK-dependent anti-inflammatory effects	[66]
Lung transplant	Rat	250–500 ppm	Improved histology, reduction of IRI and pro-inflammatory cytokines (i.e., IL-6); p38 MAPK dependent	[104,105,106]
Kidney transplant	Rat	20 ppm; 250 ppm CO	Improved graft function, reduction of IRI and pro-inflammatory cytokines, improved blood flow. Reduced chronic allograft nephropathy	[108,109,110]
Acute compartment syndrome	Pig	CORM3	Reduced tissue injury, apoptosis, leukocyte activation.	[122]

Anti-inflammatory effects of iCO at 250 parts per million (ppm) were also demonstrated in a mouse model of LPS-challenge (endotoxemia) [58], whereby administration of iCO reduced circulating pro-inflammatory cytokines (i.e., TNF-α, IL-1β, IL-6) and concomitantly increased the anti-inflammatory cytokine IL-10, reduced organ injury, and prolonged survival [58,114]. These effects were primarily associated with modulation of p38 MAPK activity [58]. However, it should be noted that p38 MAPK is not a primary target of CO, but rather a secondary target whose modulation occurs as the product of CO activation of primary receptors/targets (i.e., mitochondria or sGC), though the proximal target remains unclear. Additionally, genetic studies implicated key cellular transcription factors: Jun NH_2_-terminal kinase (JNK) and heat shock factor (HSF1) activities in these responses [114,115]. The anti-inflammatory protection against LPS-induced organ injury conferred by CO was also observed in association with impaired pulmonary iNOS expression and activity, and elevated hepatic iNOS expression and activity [116]. Taken together, these studies were suggestive of multiple mechanisms by which CO modulates anti-inflammatory signaling in animal models of inflammatory injury.

Anti-inflammatory effects were observed in a non-human primate (NHP) model of lung inflammation [95]. CO exposure following LPS inhalation decreased TNF-α release in bronchioalveolar lavage (BAL) fluid, in addition to reducing pulmonary neutrophil influx at the highest concentration (500 ppm). The therapeutic efficacy of CO in this model required relatively high doses that resulted in high CO-Hb levels (>30%), a level that is tolerated by rodents, but which would represent a hazard to humans. This study was the first to examine the therapeutic index and dose-response relationships of CO therapy in NHP in an acute inflammation model [95]. Anti-inflammatory effects of CO have been demonstrated in pre-clinical models of LPS-induced injury employing higher mammals. In the pig model, CO reduced the development of disseminated intravascular coagulation and inhibited serum levels of the pro-inflammatory cytokine IL-1 in response to LPS, whereas upregulated IL-10 levels [94].

***Acute lung injury (hyperoxia):*** Anti-inflammatory effects were observed in mice treated with iCO prior to injurious high O_2_ exposure (hyperoxia) exposure, a model of ALI. Genetic studies, using mice deficient in the mitogen activated protein kinase kinase-3 (MKK3)/p38 MAPK3 as well as in vitro studies using chemical inhibitors of p38 MAPK, demonstrated that the protective effects of CO in high oxygen toxicity were dependent on p38 MAPK [59,60].

***Ventilator-induced lung injury:*** The potential for protective effects of CO have also been evaluated in a clinically relevant model of sterile inflammation induced by mechanical ventilation which causes ventilator-induced lung injury (VILI) in rodents [101,102]. Rats ventilated with an injurious (high tidal volume) ventilator setting in combination with LPS injection, exhibited lung injury. The inclusion of CO (250 ppm) during mechanical ventilation reduced inflammatory cell infiltration, decreased TNF-α and increased IL-10 in BAL fluid [103]. iCO application conferred tissue protection in a mouse model of VILI, at moderate tidal volume ventilation [101,102]. CO reduced pro-inflammatory cytokine and chemokine production and prevented lung injury during ventilation, as evidenced by histology, and reduction of BAL protein and cell counts, lung neutrophil recruitment, and pulmonary edema [101,102]. Genetic studies further suggested that the effects of CO were associated with the activation of the plasma membrane protein caveolin-1 [101], and with upregulation peroxisome proliferator-activated receptor (PPAR)-γ, an anti-inflammatory transcriptional regulator in the lung, as well as inhibition of the pro-inflammatory factor Egr-1 [102]. These studies, taken together, suggest that CO may protect the lung in animal models of VILI. More studies are required to determine the underlying mechanisms of the therapeutic effectiveness of CO in rodent VILI models, and whether these observations may be translatable to humans.

***Pneumonia-induced ALI***: Recent studies have demonstrated that low-dose iCO accelerates resolution of ALI in a clinically relevant baboon model of pneumococcal pneumonia. The application of iCO (200 ppm for 60 min) at 48 h following *S. pneumoniae* inoculation significantly attenuated histological evidence of ALI at eight days, with evidence of enhanced mitochondrial biogenesis in alveolar type 2 epithelial cells and macrophages, and systemic antioxidant effects with increases in SOD2 expression in the kidney [96]. The CO-Hb levels achieved in this study were 6–10% [96], which is in the range of levels approved for human studies [123]. Application of iCO during pneumonia-infection also reduced pro-inflammatory urinary cysteinyl leukotrienes and improved levels of circulating specialized pro-resolving mediators (SPMs), including eicosapentaenoic acid-derived E-series resolvins (RvE) and lipoxins [70]. These results suggested that altered SPM profiles during pneumonia can be partially restored with iCO therapy [70].

***Hemorrhagic shock:*** Hemorrhagic shock and resuscitation (HSR) can cause pulmonary inflammation that induces ALI. A series of studies have shown the potential for protective anti-inflammatory effects of iCO or CORM treatment in protection against ALI and other injuries secondary to HSR. In the rat model, HSR was shown to cause ALI and pulmonary edema, cellular infiltration and hemorrhage, upregulation of inflammatory gene expression and increased lung cell apoptosis. iCO administration after resuscitation significantly prevented ALI, and reduced markers of inflammation and apoptosis, without affecting hemodynamic status or tissue oxygenation [97,98]. In animal models of HSR, therapeutic application of CORM-3 also alleviated HSR-induced ALI and pulmonary edema. CORM-3 also inhibited HSR-dependent upregulation of pro-inflammatory mediator genes (i.e., TNF-α, iNOS, and IL-1β) and expression of IL-1β and MIP-2. The expression of anti-inflammatory IL-10 was induced by CORM-3, which also inhibited lung cell apoptosis. The protective effects of iCO against HSR-induced ALI were associated with upregulation PPAR-γ. The study concluded that CORM-3 ameliorated HSR-induced lung injury via anti-inflammatory and anti-apoptotic effects, without detrimental effects on oxygenation and hemodynamics [99].

***ARDS:*** Acute respiratory distress syndrome (“ARDS”) is a type of respiratory failure characterized by widespread inflammation in the lungs which has a high (~40%) mortality rate [120,121]. The high morbidity and mortality of ARDS reflect the inefficacy of currently available diagnostic markers and therapeutic modalities [124,125,126]. To date, there are few studies examining HO-1 regulation in the pathology of clinical ARDS. Concentrations of HO-1 protein were elevated in lung tissue and in BAL fluid from patients with ARDS compared with controls. Levels of HO-1 protein in BAL fluid from patients with ARDS correlated with changes in the concentrations of ferritin and the iron saturation of transferrin but negatively correlated with concentrations of bleomycin-detectable iron [127]. Significantly elevated concentrations of HO-1 staining in cell types expressing this protein were detected in patients with ARDS, compared with concentrations in the same cells taken from controls undergoing lung resection [127]. Deregulated iron metabolism and biodistribution have also been implicated in the pathogenesis of ALI/ARDS [128]. These preliminary studies suggest modulatory or adaptive roles for HO-1 in human clinical ARDS and warrant further investigation.

***Malaria-induced lung injury (MA-ALI-ARDS):*** Malaria is a serious parasitic disease with a high incidence of mortality. Malaria associated (MA)-ALI/ARDS is one of the major clinical complications of severe malaria, which is characterized by a high mortality rate and can occur even after therapeutic interventions. DBA/2 mice infected with *Plasmodium berghei* ANKA (PbA) develop symptoms of ALI/ARDS that mimic the human syndrome, including pulmonary edema, hemorrhaging, pleural effusion, and hypoxemia. In models of MA-ALI-ARDS, in which animals are infected with murine *Plasmodium*, the induction of HO-1 was found to be protective against severe malaria complications, including the development of experimental cerebral malaria (ECM) and MA-ALI/ARDS [12,117,118]. These protective effects have been attributed to the degradation of free heme, a putative pro-inflammatory agent in malaria, and the endogenous production of CO, which can confer systemic anti-inflammatory effects [117]. Increased lung endothelial permeability and upregulation of vascular endothelial growth factor (VEGF) and other pro-inflammatory cytokines were associated with MA-ALI/ARDS and were inhibited by HO-1 induction. In severe malaria in humans, elevated HO-1 levels were found in inflammatory cells and were associated with neutrophil activation and excessive levels of inflammation [12,119].

Additionally, mice were found to be protected against malaria after treatment with either CORMs or with iCO [12,120,121]. CO prevented PbA-induced ALI in a DBA/2 mice model [119]. Furthermore, iCO can confer protection in a model of experimental cerebral malaria (ECM), following PbA infection [118]. CO prevented blood-brain barrier disruption, brain microvasculature congestion and neuroinflammation, including CD8(+) T-cell brain sequestration [117]. CO also reduced oxidation of free hemoglobin, and prevented heme release, which were associated with its anti-malarial effects [118]. The novel CORM compound ALF-492, a derivative of CORM-2, was found to protect against PbA-induced ECM, by a mechanism dependent on CO release and secondary induction of HO-1 [121]. When used in combination with the antimalarial drug artesunate, ALF-492 was an effective adjunctive and adjuvant treatment for ECM, conferring protection after onset of severe disease [121]. These studies suggest that modulation of the HO-1/CO system, in particular, pharmacological application of CORM, can be an effective therapy for MA-ALI and ECM associated with *Plasmodium* infection, and potentially enhance the efficacy of other anti-malarial drugs when used in combination therapies [12].

***COVID-19 ARDS:*** The current SARS-CoV-2 pandemic, which causes COVID-19 disease, has caused an urgent search for therapies to reverse the debilitating effects of this disease. Early studies indicate that a significant number of SARS CoV-2 infections progress to ARDS (“SARS-CoV-2 ARDS”), with resulting mortality rates for patients who do observed as high as 50%, depending on age, underlying diseases, and other factors [129,130]. In addition, health care and economic burdens associated with SARS CoV-2 ARDS care will challenge both public and private healthcare delivery systems worldwide, which emphasize the urgent need to identify and immediately implement therapeutic solutions [131]. To date, several authors have proposed to initiate studies on the relevance of HO-1, as an endogenous protective mechanism, in COVID-19 disease [132,133]. Similar to observations derived from sepsis models, the authors hypothesized that an accumulation of free heme promotes inflammatory responses including adhesion molecule expression, leukocyte recruitment, vascular permeabilization, platelet activation, complement activation, thrombosis, and fibrosis. Thus, the antioxidative and heme degrading functions of HO-1 are believed to ameliorate such conditions. To date little research has been generated in this domain. Functional barriers to these studies include difficulty in establishing COVID-19 models in mice and higher animals, regulatory hurdles, and infectious disease risk. Nevertheless, the research community cautiously awaits further investigation in this area.

***Polymicrobial sepsis:*** Protective anti-inflammatory functions of HO-1 have been demonstrated in models of polymicrobial sepsis [9,79,134]. When challenged with the cecal ligation and puncture (CLP) method to induce polymicrobial sepsis, HO-1-deficient mice (*Hmox1*^-/-^) were more susceptible to the lethal effects of CLP relative to wild type mice. The HO-1-deficient mice also displayed increased levels of free circulating heme and reduced levels of the heme binding protein hemopexin, which were associated with increased susceptibility to sepsis-induced mortality [9]. Targeted over-expression of HO-1 to smooth muscle cells and myofibroblasts of blood vessels and bowel protected against sepsis-induced mortality associated with Gram positive bacterial infection and enhanced bacterial clearance by augmenting phagocytosis and endogenous antimicrobial responses [100]. These studies suggested that endogenous heme is a deleterious factor that is associated with injurious tissue responses and increased inflammation in sepsis. Free heme has also been shown to impair bacterial phagocytosis and phagocyte migration in Gram negative sepsis [135], which may be alleviated by the heme clearance function of HO-1.

In contrast, heme has been used as a therapeutic conditioning agent, which can provide protection by inducing HO-1 expression in tissues prior to injury. Heme pre-treatment was shown to protect mice from lethal endotoxemia and sepsis induced by LPS or CLP, respectively, and this effect was related in part to reduction of pro-inflammatory cytokines and normalization of high mobility group box-1 (HMGB1) protein levels [79,134]. In the CLP model, heme conditioning also reduced mitochondrial fission, promoted mitochondrial quality control, and stimulated mitochondrial autophagy and biogenesis [136].

Another proposed mechanism by which CO can potentially alleviate sepsis is through the promotion of bacterial clearance, via incompletely understood mechanisms [67,79,100,137]. Application of iCO (250 ppm) either as pre-treatment or post treatment improved mouse survival in the CLP model [67]. The protective effects of CO in CLP were related to the induction of autophagy and phagocytosis, the reduction of inflammation, and enhanced bacterial clearance from organs and blood. The pro-survival effects of CO in CLP were dependent on the autophagy program, as they were reduced in mice heterozygous for genetic deletion Beclin-1, a major regulator of autophagy [67].

The pharmacological application of CORMs enhanced bacterial phagocytosis *in vivo* and rescued *Hmox1*^-/-^ mice from sepsis-induced mortality [100]. CORMs improved intestinal bacterial clearance in an *S. typhimurium* infection model [137]. In an *E. coli* infection model, endogenous HO-derived CO was associated with enhanced macrophage phagocytosis and this was shown to require inflammasome-dependent immune responses [64].

In contrast, genetic studies have revealed an overall pro-pathogenic role for NLRP3 in CLP-induced sepsis [138]. Consistent with this model, CO derived from CORM-3 has been shown to exert myocardial protection in CLP in part by downregulation of NLRP3-dependent inflammasome activation [139]. Similarly, CO derived from CORM-2 can exert protection in sepsis-induced acute AKI in rats subjected to CLP [140], as evidenced by reduced serum creatinine and blood urea nitrogen levels, reduced kidney cell apoptosis, increased survival, and decreased renal histology scores. Treatment with CORM-2 reduced TNF-α and IL-1β levels and oxidative stress, and inhibited inflammasome-associated caspase-1 activation [140]. Taken together, these experiments suggest that CO can confer multi-organ protection in sepsis, in part by improving bacterial clearance and/or modulating inflammasome dependent responses.

In human studies, arterial blood CO, bilirubin levels, and HO-1 protein expression in monocytes were found to be higher in patients with severe sepsis/septic shock than in non-septic patients, and corelated with patient survival [141].

***CO in ischemia/reperfusion injury and primary graft dysfunction***: Lung transplantation (LT), is often used for advanced stage lung diseases, yet its success has been compromised by the high incidence of acute graft rejection. Primary graft dysfunction (PGD) is a complication of LT that arises within 72 h of surgery and is responsible for early morbidity and mortality associated with LT. PGD occurs as the consequence of IRI of the organ incurred during the transplantation process [142]. The mechanism(s) of IRI involve neutrophil influx at the site of tissue injury, release of pro-inflammatory mediators, increased ROS production, and pro-inflammatory cytokines, which promote cellular injury. PGD is associated with activation of an inflammatory cascade, which can predispose the graft to chronic rejection [143].

Preclinical data have shown that the anti-inflammatory and antiapoptotic properties of iCO can confer potent cyto-protection in a rat model of LT [104]. In a study involving orthotopic left lung transplantation in rats, transplanted lungs displayed severe intra-alveolar hemorrhage, infiltration of inflammatory cells, and intravascular coagulation. In the presence of iCO (500 ppm), the histology of transplanted lungs showed marked preservation [104], with reduced apoptotic cell death and inflammatory cytokines production compared with the transplanted lungs of non-CO-exposed recipients. Further studies have confirmed beneficial effects of iCO in a rat model of LT [105,106,107]. Kohmoto et al. demonstrated that exogenous low-dose CO treatment of donors and recipients can prevent lung IRI and significantly improve function of lung grafts after extended cold preservation and transplantation [105,106,107]. This group also demonstrated that exposure of donor and recipients to 250 ppm CO via inhalation is anti-inflammatory and preserves normal endothelial cell and pneumocyte ultrastructure in lung grafts [106]. CO improved pulmonary vein pO_2_ in the graft after transplantation, and significantly inhibited neutrophil migration into the graft, and preserved lung ultrastructure [106]. Low concentration CO has been found to exert protective effects in IRI associated with transplantation of other organs, including kidney [108,109,110,144], as well as liver, pancreas and intestine, as described elsewhere [111]. In pigs, CO derived from CORM-3 mitigated skeletal muscle IRI associated with acute compartment syndrome [122].

## 3. Therapeutic Modulation of HO-1 in Human Disease

The therapeutic modulation of HO-1 for amelioration of human diseases has been proposed by many investigative groups. Metalloporphyrins (i.e., tin and zinc protoporphyrin) which as competitive inhibitors of HO activity, have been extensively studied as an alternative to phototherapy for BR accumulation in neonatal jaundice [145]. More selective HO-1 inhibitors have been described [146,147]. Novel genetic knockdown methods using the CRISPR/Cas9 system have proven effective at inhibiting HO-1 expression in vitro, albeit with noted effects on cell growth, proliferation, and sensitivity to oxidative stress [148].

The prospects of gene therapy approach to drive HO-1 expression are limited by current technology advances in this domain. Since HO-1 generates several reaction products with multiple effector functions, the effects of HO-1 gene delivery will likely incur pleiotropic and off-target effects. These efforts may benefit from highly targeted cell-or tissue-specific approaches to gene delivery, as recently demonstrated in adipocytes [149]. Natural antioxidants can be used to target systemic HO-1 expression, and the mechanisms of action of many such natural plant derived substances including polyphenolic antioxidants, has been related in part to enhanced HO-1 expression [150,151,152]. Use of these compounds orally, will likely be non-selective. Synthetic inducers of HO-1 such as cobalt-protoporphyrin may also have pleiotropic effects and unsuitable toxicity profiles for human use/consumption. Dimethylfumarate, a drug developed for multiple sclerosis, activates Nrf2-dependent antioxidant responses, and may have further potential for clinical use [153]. Among the natural HO-1 enzymatic byproducts, iCO remains closer to therapeutic development, as discussed below.

## 4. Inhaled Carbon Monoxide (iCO): Summary of Current Clinical Progress

Based on a solid foundation of preclinical studies that were performed over the last two decades in rodents and higher animals (including swine and NHPs), ongoing investigations continue to seek the development of inhaled CO (iCO) as a therapeutic agent for critical care medicine applications and other human diseases. Despite initial promise of preclinical studies, therapeutic development has yet to deliver an immediate breakthrough, due to funding, regulatory hurdles, and public misconceptions despite the demonstration of safety [154,155]. Nevertheless, completed clinical trials to date have demonstrated that therapeutic application of iCO in humans is well-tolerated at low dose, and feasible [156,157]. Early trials of patients with chronic lung disease demonstrated that iCO was safe and did not cause adverse events [157]. A recent multicenter phase IIa, double-blinded, sham-controlled, clinical trial was completed, in which idiopathic pulmonary fibrosis patients were randomized to receive iCO or medical air for 12 weeks [158]. The study reported no differences in physiological measures, incidence of acute exacerbations, hospitalization, death, patient-reported outcomes, or secondary endpoints. Furthermore, no differences were reported in the distribution of adverse events. This study concluded that iCO can be safely administered to IPF patients [158].

A phase I dose escalation trial was recently performed to assess feasibility and safety of low-dose iCO administration in patients with sepsis-induced ARDS. Study subjects (*n* = 12) were randomized to iCO or placebo air 2:1 in two cohorts. In this trial, patients were administered iCO (100–200 ppm) or placebo air for 90 min for five days [123]. No participants exceeded a CO-Hb level of 10%, and no adverse events were reported. iCO-treated participants (100–200 ppm) displayed significant increases in COHb (up to 5%) compared with placebo-treated subjects. These studies reported that the Coburn–Forster–Kane (CFK) equation was highly accurate at predicting CO-Hb levels in patients exposed to 200 ppm CO. The study concluded that precise administration of low-dose iCO is safe and feasible in patients with sepsis-induced ARDS. Furthermore, circulating mtDNA levels were reduced in the iCO treatment groups. The study provided a precedent for ongoing Phase II studies (NCT03799874) and planned future efficacy trials [123].

## 5. Conclusions

From a biological perspective, the HO-1 field continues to draw worldwide interest, with a multiplicity of biological roles recognized for HO-1 and its reaction products, from regulation of inflammation to immune system modulation. Mitochondrial regulation has emerged as a principal target of CO. A number of strategies have been explored toward harnessing the therapeutic potential of HO-1/CO in diseases such as ALI/ARDS, and related conditions such as VILI, sepsis, and viral or bacterial pneumonias. Application of CORMs and iCO remain at the forefront of therapeutic development efforts. CORMs development continues with new compounds development and preclinical studies in a wide variety of indications [25,159,160,161,162] yet has met limitations with identifying compounds with toxicity profiles suitable for human clinical use [154,155]. The principal barriers to the use of iCO are several fold. (i) Although proof-of-concept has been achieved in animal models, efficacy in human clinical trials has yet to be demonstrated, although current trials have demonstrated safety and feasibility. (ii) The general public has a maladaptive perception of CO as an absolute poison, due to its visible toxicity and lethality from high dose exposures, which preclude the general acceptance of a potential therapeutic window. (iii) Regulatory agencies may set upper limits for testing, though there are no universal guidelines. Recently completed studies that gained FDA approval set a target CO-Hb of 6–8%, with an upper limit not to exceed 10% [123]. Thus, if CO has a therapeutic value that can be exploited for clinical use, based on extensive preclinical evidence to this effect, then there is a need for the development of safe and efficacious delivery mechanisms that circumvent toxicological and regulatory concerns. Research and development in this area are expected to continue for the foreseeable future.
